# A blood-free method of performing slit-skin smears

**DOI:** 10.1016/j.jdin.2024.01.008

**Published:** 2024-04-23

**Authors:** Matthew J. Verheyden, Margot J. Whitfeld, Meciusela Tuicakau, Antoine Bertolotti

**Affiliations:** a Department of Dermatology, the Sutherland Hospital, Caringbah, New South Wales, Australia; b Sydney Medical School, University of Sydney, Sydney, New South Wales, Australia; c Skin Hospital, Sydney, New South Wales, Australia; d Department of Dermatology, PJ Twomey Hospital, Suva, Fiji; e CIC-INSERM 1410, Department of Dermatology, University Hospital of Reunion Island, Saint-Pierre, Reunion, France; f Kirby Institute, University of New South Wales, Sydney, New South Wales, Australia

**Keywords:** general dermatology, infectious diseases, leprosy, medical dermatology

## Challenge

Slit-skin smears are a cost-effective, readily accessible technique used in the diagnosis and monitoring of patients with leprosy.^1^ The conventional approach involves a superficial incision using a number 15 scalpel blade in a “cold” area of the body (earlobes, elbows, etc) to obtain bloodless interstitial fluids for analysis. A commonly encountered issue when using a scalpel blade is accidental deeper incisions, leading to unintended bleeding. Excessive blood may compromise the interpretation of the slide. Deeper incisions may also lead to prolonged bleeding and delayed hemostasis rather than the crust resulting from a more superficial incision. Moreover, the use of a scalpel blade poses risk of sharps injuries to the patient, proceduralist, and other staff involved in the procedure.

## Solution

**Fig 1:**
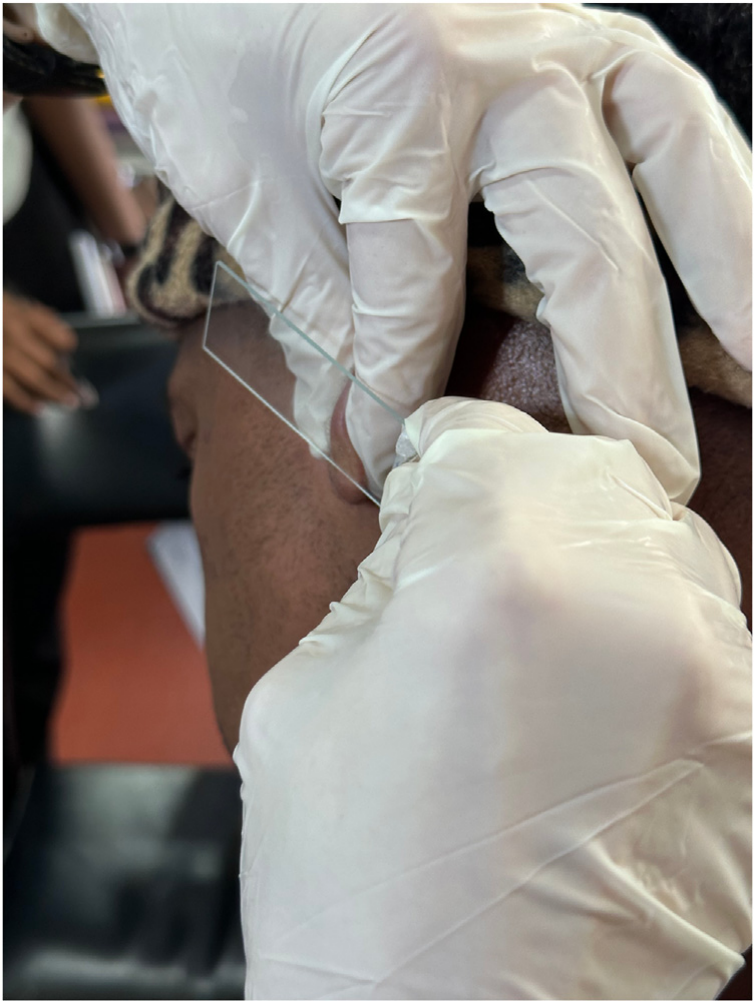
Specimen collection.

**Fig 2:**
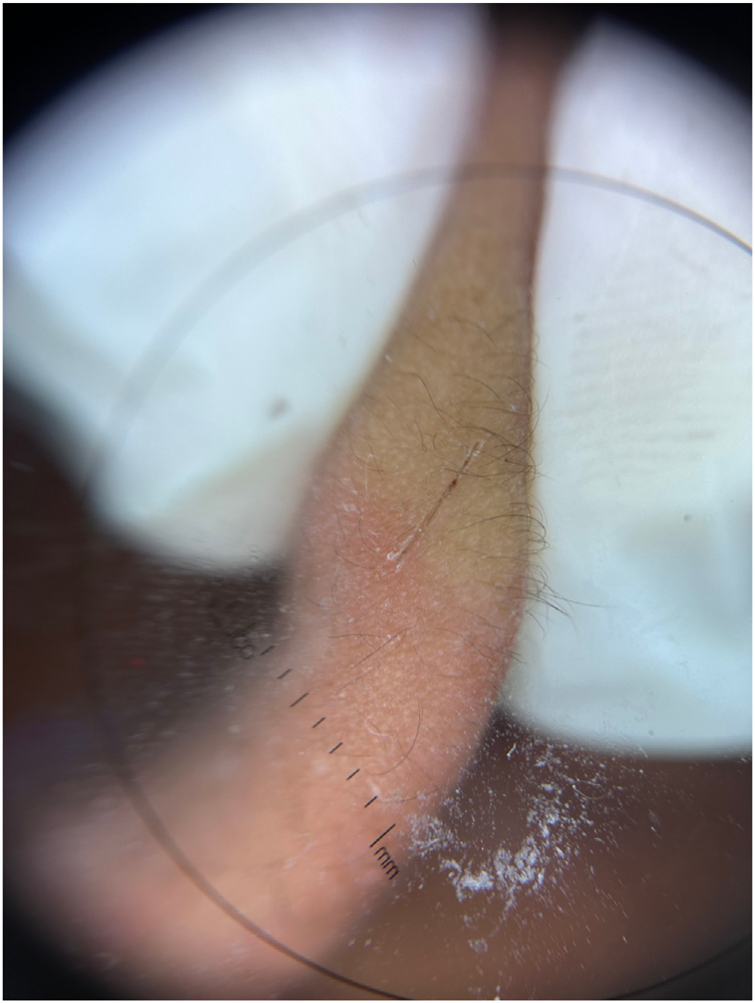
Dermatoscopy of crusting ear lobe after incision.

The technique mitigating these problems is to use a blunt pair of sterilized surgical scissors in place of a scalpel blade. We demonstrate the application of this technique on the earlobe of a patient with leprosy (Video 1, available via Mendeley at https://data.mendeley.com/datasets/zbndr4vdjz/1). Firm pressure is applied with the thumb and forefinger of the nondominant hand on either side of the ear lobe, then with the dominant hand the cutting edge of the scissors are applied with gentle pressure and pulled along the length of the blade to make a superficial incision. Pressure is maintained on the earlobe while the glass slide is used to collect the bloodless interstitial fluid (Fig 1). The specimen can then be air-dried at room temperature and processed as usual. When performed correctly, a crust forms promptly on the linear incision after completing the technique, as demonstrated by dermatoscopy (Fig 2). Although not eliminated, the risk of sharps injury is diminished when compared with the conventional approach using a scalpel blade. In resource-limited settings, access to sterilized surgical scissors is more readily available and more cost-effective than scalpel blades. The procedure’s ease of execution and reduced risks make it an attractive alternative to the conventional scalpel blade method.

## Conflicts of interest

None disclosed.
